# Phylogenetics and biogeography of a spectacular Old World radiation of butterflies: the subtribe Mycalesina (Lepidoptera: Nymphalidae: Satyrini)

**DOI:** 10.1186/1471-2148-10-172

**Published:** 2010-06-10

**Authors:** Ullasa Kodandaramaiah, David C Lees, Chris J Müller, Elizabeth Torres, K Praveen Karanth, Niklas Wahlberg

**Affiliations:** 1 Department of Zoology, Stockholm University, 106 91 Stockholm, Sweden; 2 Department of Entomology, Natural History Museum, London, UK; 3 Centre de Recherche d' Orléans, INRA, UR 633 Zoologie Forestière, F-45075, Orléans, France; 4 Molecular Ecology Lab, Department of Biological Sciences, Macquarie University, Sydney, NSW 2109, Australia; 5 Department of Biological Sciences, California State University, Los Angeles, CA 90032 USA; 6 Center for Ecological Sciences, Indian Institute of Sciences, Bangalore, India; 7 Laboratory of Genetics, Department of Biology, University of Turku, 20014 Turku, Finland

## Abstract

**Background:**

Butterflies of the subtribe Mycalesina (Nymphalidae: Satyrinae) are important model organisms in ecology and evolution. This group has radiated spectacularly in the Old World tropics and presents an exciting opportunity to better understand processes of invertebrate rapid radiations. However, the generic-level taxonomy of the subtribe has been in a constant state of flux, and relationships among genera are unknown. There are six currently recognized genera in the group. *Mycalesis*, *Lohora *and *Nirvanopsis *are found in the Oriental region, the first of which is the most speciose genus among mycalesines, and extends into the Australasian region. *Hallelesis *and *Bicyclus *are found in mainland Africa, while *Heteropsis *is primarily Madagascan, with a few species in Africa. We infer the phylogeny of the group with data from three genes (total of 3139 bp) and use these data to reconstruct events in the biogeographic history of the group.

**Results:**

The results indicate that the group Mycalesina radiated rapidly around the Oligocene-Miocene boundary. Basal relationships are unresolved, but we recover six well-supported clades. Some species of *Mycalesis *are nested within a primarily Madagascan clade of *Heteropsis*, while *Nirvanopsis *is nested within *Lohora*. The phylogeny suggests that the group had its origin either in Asia or Africa, and diversified through dispersals between the two regions, during the late Oligocene and early Miocene. The current dataset tentatively suggests that the Madagascan fauna comprises two independent radiations. The Australasian radiation shares a common ancestor derived from Asia. We discuss factors that are likely to have played a key role in the diversification of the group.

**Conclusions:**

We propose a significantly revised classification scheme for Mycalesina. We conclude that the group originated and radiated from an ancestor that was found either in Asia or Africa, with dispersals between the two regions and to Australasia. Our phylogeny paves the way for further comparative studies on this group that will help us understand the processes underlying diversification in rapid radiations of invertebrates.

## Background

Knowledge of phylogenetic relationships among the species comprising a rapid radiation has proved invaluable for detailed investigations into the processes and patterns of their diversification. There are still relatively few studies aimed at understanding the mechanisms of radiations for invertebrates, even in popular groups such as butterflies which feature prominent model-organisms in evolutionary biology [1]. It is the lack of robust phylogenies for such groups that has imposed a crucial impediment for comparative analyses. Among butterflies, a phylogenetic perspective has been applied to a number of radiations (e.g. [2-6]). However, few butterfly groups can compare with the mycalesine radiation (Nymphalidae: Satyrinae: Satyrini: Mycalesina) in terms of diversity of species and geographic sweep. They have been acclaimed as one of the most spectacular butterfly radiations, comprising more than 270 species usually placed in six genera [7,8]. Mycalesines are found across the Old World tropics in both forested and open habitats and are characterized by high levels of endemicity throughout their range. Unlike other butterfly radiations, which typically peak in diversity within a single zoogeographic region (e.g. *Arhopala *in SE Asia), all major palaeotropical regions - Madagascar, Africa, the Indian subcontinent, Indo-China, the larger islands of South-East Asia (notably Sulawesi), New Guinea and the Solomon Islands are each represented by a species-rich mycalesine fauna. The Indo-Australian, Afrotropical and Madagascan regions have roughly equal numbers of species [7]. This group of butterflies thus presents an exciting opportunity to understand their diversification within a phylogenetic framework.

The level of diversity in fundamental body plan and in larval and adult feeding biology is relatively modest compared to other butterfly groups with similar species richness. This relative morphological and behavioural conservatism contrasts sharply with a spectacular array of scent organs found in males and some striking differences in expression of wing ocelli and colour patterns [7,9]. The existing generic classification scheme has been based mostly on limited morphological characters such as the presence of hairy eyes (interommatidial setae), forewing venation [9-11] or scent organs [12], which have been considered inadequate character sets for resolving their systematics [13-15]. The circumscriptions of genera have been in a constant state of flux, with early revisions for instance [12] and [16] for Asian and African species respectively, and several more recent local revisions based on morphology.

Despite work on regional mycalesine faunas, there has been no coherent attempt to date to classify the entire group. The need for robust phylogenetic hypotheses from molecular data to resolve this issue has been stressed [8,17]. A study on the mycalesines of Madagascar (also including a few African species; [8]) using molecular data from two mitochondrial genes (Cytochrome oxidase II and Cytochrome b) reported that the majority of Madagascan genera were not monophyletic, corroborating results from a previous morphological study [7]. Accordingly, the pre-existing genera *Henotesia*, *Admiratio *and *Masoura *were subsumed under *Heteropsis *as sub-genera, while *Houlbertia *was sunk completely [8,18]. The Afrotropical region now consists of three genera *Heteropsis*, *Hallelesis *and *Bicyclus *[9,19]. The genus *Heteropsis *Westwood (1850) comprises around 81 known species of which 46 are described and a further ca. 24 undescribed species are known in the Malagasy Region [7,18,20]. About 12 *Heteropsis *species are distributed in continental Africa [21-24] whilst *Hallelesis*, with two species, is confined to West and Central Africa [25]. *Bicyclus *consists of ca. 80 species in mainland Africa [9] with one species, *B. anynana*, also found in the Comoros [26]. Species-level relationships within the genus were investigated in a molecular study [27], for which data from three other mycalesine genera were used to root the tree. Although *Bicyclus *was eventually recovered as a clade, their sampling of other genera was too poor to establish firm support for its monophyly.

The remaining three genera (*Mycalesis *Hübner, 1818, *Lohora *Moore, 1880 and *Nirvanopsis *Vane-Wright, 2003) are found in the Indo-Australian tropics. *Mycalesis *is the most species-rich among current mycalesine genera with estimates of the number of species ranging from 87 to over 100 [28-31]. This genus is almost ubiquitously distributed in the Indo-Australian region ranging from Sri Lanka and India in the West, across Indo-China, South-East Asia and New Guinea, to North-East Australia and the Solomon Islands in the East. *Lohora *and *Nirvanopsis *are endemic to Sulawesi. *Lohora *contains 17 species and *Nirvanopsis *was for a long time considered to be monobasic (previously as *Nirvana*), but now includes a recently described species, *N. susah. *It has been suggested that *Mycalesis *is probably paraphyletic with respect to *Lohora *[31], and that *Nirvanopsis *might belong within *Lohora *[17]. *Mycalesis *was considered closely related to another Indo-Australian mycalesine genus *Orsotriaena*, but this has been refuted by recent molecular studies [32,33]. *Mycalesis *differs from *Bicyclus *by the presence of hairy eyes; dense interommatidal setae are absent in the latter [14]. *Heteropsis *also has hairy eyes [7] but differs from *Mycalesis *in details of wing venation and for most species, male genitalia [7]. *Mycalesis *was divided into several genera [12], and into species groups by [34] and [29]. Additional file 1 provides more information on taxonomical revisions and groupings of Asian and Australasian species.

The monophyly of genera and the relationships among them have strong implications for our understanding of their global diversification. Miller, whose study [11] was largely based on an examination of leg morphometrics, speculated that mycalesines started diverging in the Oriental region from an ancestor which was initially derived from Neotropical satyrines. According to his scenario, Africa was colonized twice, once by a naked eyed ancestor (leading to *Bicyclus *and *Hallelesis*) and by a hairy eyed ancestor that eventually went on to disperse into Madagascar (leading to *Heteropsis*) and went extinct in Africa. Miller further speculated that the hairy-eyed mycalesines dispersed into the Australasian region. Based on their tree where the African *Heteropsis *was nested within the Madagascan clade, Torres and colleagues [8] suggested an alternate scenario where Africa was colonized at least once from Madagascar.

Hostplant records for this group are scarce; the known records are mainly from Poaceae, but also from Cyperaceae, Marantaceae and Zingiberaceae [35]. Most regional groups tend to be restricted to the forested tracts of lower altitudes [10,36,37], whereas in some regions such as Madagascar there is elevational zonation along the entire forest gradient [38]. Mycalesines are generally low flying butterflies with weak to moderate dispersal abilities [8,25,39] (although the *Heteropsis *subgenera *Masoura *and *Admiratio *are canopy species, as is *Nirvanopsis*) [40,41]. This low dispersal ability along with habitat and bioclimatic fidelity renders several species endemic to narrow regions [10,40]. With some exceptions, species are dull and cryptically coloured, bearing a postdiscal series of eyespots (ocelli) on the dorsal and ventral surfaces that are sometimes not expressed on some wing surfaces or in both sexes. Species that experience defined periods of wet and dry seasons have a corresponding dry- and wet-season form [42]. This polyphenism is characterized by the reduction of the ventral eyespots in the dry season morph.

Mycalesines have been used extensively in various ecological (e.g. [12,40,43,44]) and evolutionary studies (e.g. [45-50]). *Bicyclus *in particular has carved a niche for itself as a model organism in evolutionary biology, with the eyespots in *B. anynana *having been the focus of innumerable evo-devo studies (e.g. [51-54]). Almost all species of Mycalesina possess eyespots, but, again, the lack of robust phylogenies has hindered comparative studies within a phylogenetic framework; the two studies on *Bicyclus *[50], and [55], are the only such studies so far.

No molecular study has incorporated sufficient species from all mycalesine genera for a rigorous test of their reciprocal monophyly. The two studies [8,27] were focused on regional mycalesine faunas. The former study did include a sample of the type species of *Mycalesis*, *M. francisca*, but found no support for its placement. These authors concluded that denser taxon sampling was necessary to elucidate generic level relationships within the group. In this study, we attempt to infer the phylogeny of Mycalesina using sequence data from three genes. We also include 42 species of *Mycalesis *and seek to identify major lineages within the genus and their relationships. We estimate lineage divergence times within the group and attempt to reconstruct events in its biogeographic history.

## Results

### Systematics

**Figure 1:**
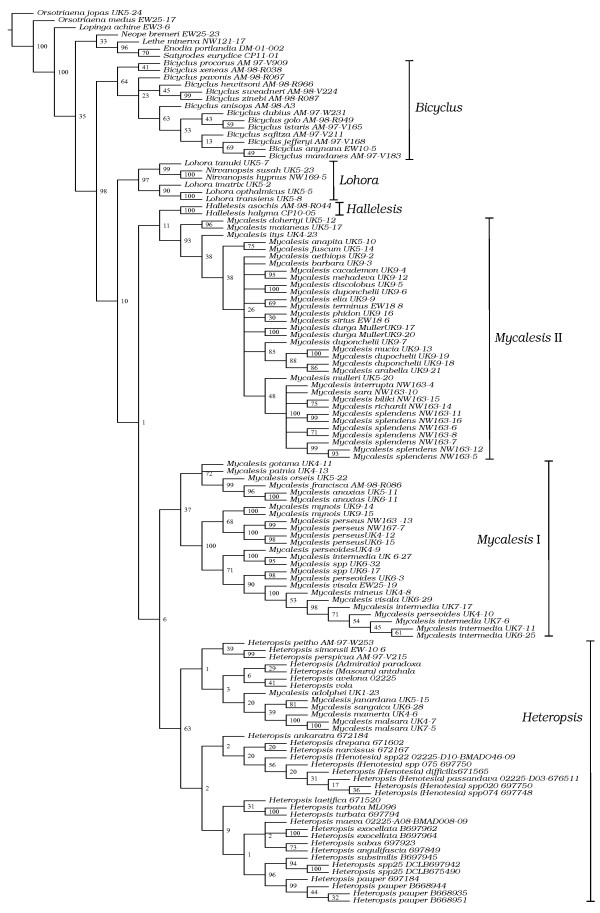
**Strict consensus topology of the 80 equally most parsimonious trees recovered in the Maximum Parsimony analyses of the combined dataset in TNT (Length = 7290)**. Numbers indicate bootstrap support for nodes. Names of the six 'stable clades' identified in this study are indicated next to the taxon names (see Results section).

**Figure 2:**
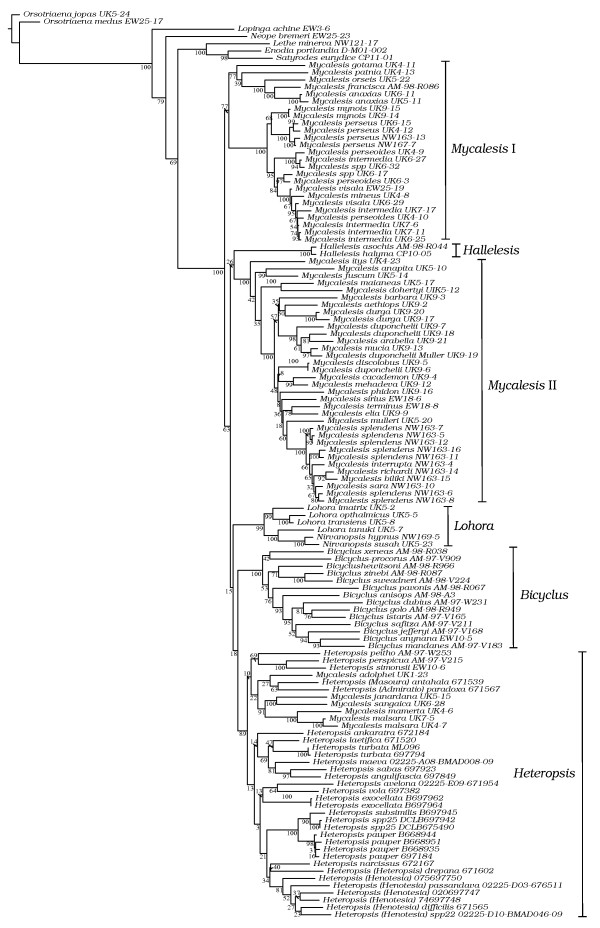
**Maximum Likelihood topology recovered from the RAxML analysis of the combined dataset**. Numbers indicate bootstrap support for the nodes to the right. Names of the six 'stable clades' identified in this study are indicated next to the taxon names (see Results section).

**Figure 3:**
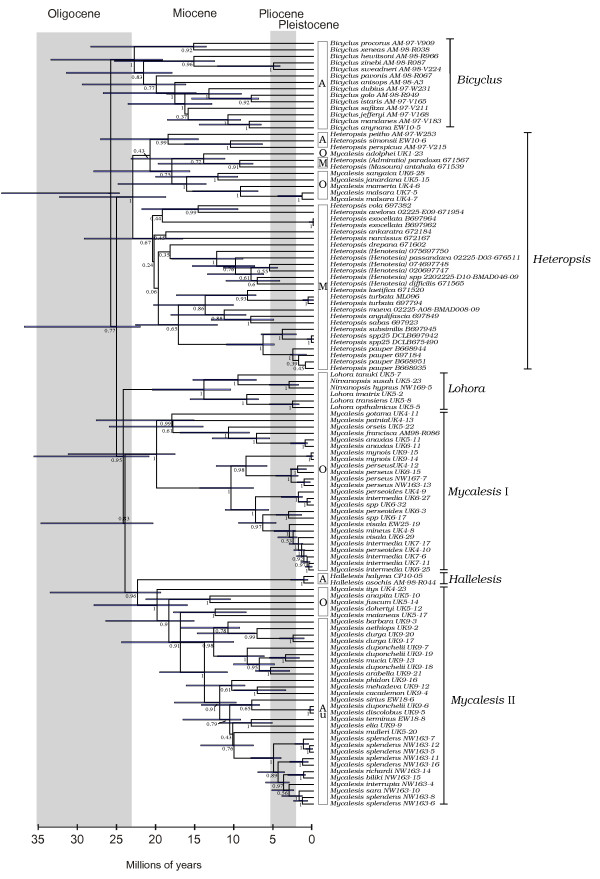
**Ultrametric tree resulting from the Bayesian analysis of the combined dataset in BEAST**. Numbers to the left of nodes are posterior probability values. Horizontal bars are 95% confidence intervals. Distributions of the taxa are shown to the left of the taxon names, as follows - Africa (A), Oriental (O), Madagascar (M) and Australia (Au). Names of the six 'stable clades' identified in this study are indicated next to the taxon names (see Results section).

The combined dataset included 3139 base pairs (bp) from 125 samples including seven outgroups. The MP (Maximum Parsimony) analysis of the combined dataset resulted in 80 equally most parsimonious trees, the strict consensus of which is shown in Fig 1. The ML (Maximum Likelihood) analysis on the same dataset resulted in a topology that differed at several nodes (Fig 2). However, these differences were mainly in the basal nodes that were weakly supported in both analyses, and in the ML tree, preceded by short branches. Nodes that were well supported (>80% bootstrap values) in the ML analysis also figured in the MP topology, albeit with lower support in general. Both analyses supported the monophyly of Mycalesina (MP: 98% bootstrap; ML: 100% bootstrap). The BI (Bayesian Inference) tree was different to both MP and ML, but incongruence was restricted mainly to the basal part of the tree (Fig 3). Nodes supported strongly in the ML analysis were recovered with strong posterior probabilities in the BI tree. Results that are consistent across the three tree-building methods are elaborated below.

*Bicyclus*, *Hallelesis *and *Nirvanopsis *emerged as monophyletic groups in all three analyses whereas the remaining genera were either paraphyletic or polyphyletic. The monophyly of *Bicyclus *was strongly supported in the model-based analyses but only moderately so in MP. *Hallelesis *and *Nirvanopsis*, each represented by two species, were monophyletic with strong support. *Nirvanopsis *was nested within *Lohora*, rendering the latter paraphyletic. Relationships among *Heteropsis *species were broadly consistent with subgeneric groupings in [7]. A group consisting of *M. adolphei, M. janardana, M. sangaica, M. mamerta *and *M. malsara *was nested within *Heteropsis*. The remaining *Mycalesis *species clustered into two clades, but *Mycalesis *as a whole was polyphyletic.

Six higher clades within Mycalesina were common to all three analyses, each with moderate to strong support in the model-based analyses, but weaker support in MP. These will be referred to as the stable clades and have been named either with novel or available names to facilitate discussion (Figs 1, 2 and 3). Relationships within these stable clades were similar, if not congruent, between analyses (an exception is the unresolved nature of *Mycalesis *clade II in MP). Some previously recognised species groups (Additional file 1) in *Mycalesis *were also recovered as well-supported clades. The Mineus group of Evans (2-species group of Aoki and colleagues [29]; approximating to *Calysisme *of Moore plus *Jatana*) was one such group, which includes *M. mynois, M. perseus, M. mineus, M. intermedia, M. perseoides *and *M. visala*. *M. perseus *is the most widely distributed *Mycalesis *species, ranging from the Indian subcontinent to NE Australia. The other members of the Mineus group are restricted to the Oriental region. *M. patnia *('4-species' group of Aoki and colleagues [29]) was either nested within or sister to the '1-species' group (including *M. gotama, M. patnia, M. orseis, M. francisca *and *M. anaxias*; equivalent respectively to Moore's *Sadarga*, *Nissanga*, *Suralaya*, *Gareris *and *Virapa*). Members of these three species groups together formed a stable clade, which will be referred to as *Mycalesis *clade I.

The Australasian species (*M*. *barbara, M. aethiops, M. mucia, M. phidon, M. mehadeva, M. cacodaemon, M. sirius, M. duponchelii, M. discolobus, M. elia, M. terminus, M. mulleri, M. splendens, M. interrupta, M. biliki, M. richardi *and *M. sara*) in addition to the Oriental *M. dohertyi, M. maianeas, M. itys, M. fuscum *and *M. anapita *formed another stable clade, hereafter referred to as *Mycalesis *clade II (equivalent to *Mydosama *of Moore plus *Savanda*, *Nebdara*, *Satoa *and possibly *Culapa*). The Oriental genera within the clade were basal members, while the Australasian species were a strongly supported clade nested within. The other stable clades within Mycalesina are *Lohora *(*Lohora *+ *Nirvanopsis*), *Hallelesis*, *Bicyclus *and *Heteropsis *(including some species of *Mycalesis*; Fig 1, 2 &3).

ML analysis on the COI dataset recovered the six stable clades (Additional file 2). *Mycalesis *clade II was nested within *Mycalesis *clade I in the EF-1α tree, while the remaining four stable clades were retrieved (Additional file 3). The *wingless *tree was generally poorly-supported with very short branches, but recovered the *Heteropsis *and *Lohora *clades (Additional file 4). *Hallelesis *and *Bicyclus *were represented by a single species, whereas *Mycalesis *clades I and II were not monophyletic.

### Shimodaira-Hasegawa tests

The likelihood score of the ML topology in Fig 2 (In *L *= -38610.95571) was significantly higher than the "genus monophyly" topology (ln *L *= -38702.28514), wherein each genus was constrained to be monophyletic (P = 0.002, Shimodaira-Hasegawa test, Table 1). On the contrary, there was no significant difference in likelihood scores between the ML topology and the tree where *Mycalesis *clades I and II were reciprocally monophyletic.

**Table 1:** Results of Shimodaira-Hasegawa tests (see text for details)

Topology	-ln L	Difference -ln L	P value
ML	38610.96	-	-

GM	38702.298514	91.33	0.002*

MM	38624.71	13.76	0.47

* P < 0.05. ML: unconstrained ML tree in Fig 2. GM: "genus monophyly" topology where each genus was constrained to be monophyletic. MM: Topology where Mycalesis clade I and II were reciprocally monophyletic.

### 'Rogue' analysis

Each of the six stable clades was successively removed and the resulting datasets subjected to RaxML analyses. The resulting trees are summarized in Additional file 5. We found that deleting any of the clades did not increase support for basal nodes; nodes with <50% bootstrap values did not receive >50% support.

### Divergence time estimates

The dating analysis in BEAST indicates that the ancestors of the six stable clades diverged between 26 and 21 mya (Fig 3) Initial splits within the *Lohora*, *Bicyclus*, *Mycalesis *groups I and II began ca. 18-20 mya. The two species of *Hallelesis *started diverging ca. 1.5 mya.

## Discussion

Although we found several well-supported higher clades within Mycalesina that were stable with respect to the method of analysis, relationships between these clades varied considerably between the analyses. Some of the instability could perhaps be due to missing data for some taxa. However, incongruent relationships were those that were supported poorly in all analyses, which suggests that the conflict between the methods is due to poor phylogenetic signal in the dataset. We believe that the basal divergences have happened in rapid succession with little time for synapomorphic changes to accumulate in the intervening period between two successive splits [56,57]. This pattern is similar to that reported in other satyrine groups [58,59]. Increasing the number of genes has been shown to improve resolution of rapid radiations in some cases, whilst some nodes remained poorly supported despite adding data from multiple genes [60-62]. The synergistic effect of the addition of morphological data to molecular datasets has been demonstrated before [63]. However, morphology has not proved satisfactory for the higher level classification of the group and it remains to be seen whether addition of morphological characters will resolve basal relationships, though we suspect that these nodes will be difficult to resolve.

Although basal relationships are unresolved by the current data we do find several well-supported evolutionary groups. The results call for major redefinitions of mycalesine genera as well as illuminate aspects of the biogeographic history of the group.

### Systematic implications

We propose that the group of *Mycalesis *species nested within *Heteropsis *should henceforth be classified under *Heteropsis. Mycalesis *clades I and II were never sister to each other in the combined analyses, although a topology with *Mycalesis *clades I and II constrained to be sister to each other was not significantly worse than the ML topology (where they are not sister to each other). Nevertheless, we propose that members of clade I and II should be classified under two respective genera. The genetic divergence between the clades is comparable to the divergence between other genera in our study, supporting our re-classification scheme. Furthermore, the two clades almost completely allopatric, with clade I restricted to South and SE Asia, whereas clade 2 is predominantly Australasian. Moreover, even under the unlikely event that these two clades indeed turn out to be each others' sisters with strong support, our proposed classification system would still remain valid. Clade I includes the type species of the genus (*M. francisca*) and we propose that members of this clade should be classified under *Mycalesis sensu stricto*. We transfer the species in the *Mycalesis *Clade II to *Mydosama *(type species, *Dasyomma fuscum *Felder & Felder, 1860). We also subsume *Nirvanopsis *under *Lohora*.

### Biogeography

It is important to bear in mind that our divergence time estimates are based on a single secondary calibration point (from [64]) and as such are a first approximation. We here present our interpretation of the biogeographic history of the group based on these timing estimates and the strongly supported monophyletic groupings. The age of the most recent common ancestor of Mycalesina suggested by the dating analysis precludes a Gondwanan origin of the group. The most likely sister clade of the group is composed of a group of genera distributed predominantly in the Oriental and the easternmost parts of Palaearctic region [59,64]. Miller's hypothesis [11] was that the ancestor of mycalesines was of Oriental origin and the group diversified through dispersals to the Afrotropical and Australasian regions. Mycalesis clade I emerged sister to remaining mycalesine clades in the ML tree, in support of Miller's hypothesis. However, the MP and BI trees retrieved the African *Bicyclus *as sister to the rest of the mycalesines, although without strong support for the latter group. This would point to an African origin, as already shown in two other nymphalid groups, *Junonia *and *Charaxes *[6,65]. In this scenario, mycalesines started evolving in Africa, with subsequent colonization of Madagascar where there was explosive radiation of the *Heteropsis *group, and this group subsequently invaded Asia. With the poorly supported basal relationships, however, we are unable to distinguish between hypotheses of African and Asian origin. Nonetheless, it is clear that there has been at least one dispersal event between the Afro-Malagasy and Asian regions in the early evolution of the group. This colonization(s) has most likely occurred across the Arabian Peninsula, which is thought to have been covered by forests at some point during the Miocene [66,67]. The Arabian Peninsular region appears to have been important for butterflies as a corridor of 'geodispersal' (concordant dispersal of several lineages over the same route) [68] between Africa and Asia.

The grouping of some Asian mycalesines with the Afro-Malagasy genus *Heteropsis *is well supported by all combined analyses and independently by the COI and EF-1α datasets (note that the close relationship of *M. adolphei *with *Heteropsis *is also supported by morphological data, in particular the form of the male genitalia; [7,18]). This grouping suggests at least one dispersal event from the Afrotropical to the Oriental Region, as the Oriental members are derived within the *Heteropsis *group. Our trees also suggest tentatively (although lacking support for the placement of *Admiratio *+ *Masoura*) that the Madagascan mycalesines comprise two independent radiations (subgenera *Admiratio+Masoura*, and the clade including subgenera *Heteropsis+Henotesia*). Greater taxon and gene sampling (note that *Admiratio *and *Masoura *are only represented in our tree by COI) is therefore needed to establish a robust topology for *Heteropsis sensu lato *that can lead to a stronger inference of the biogeographic history of the group. It is very probable that two species (*H. comorana *and *H. narcissus*) have colonised the Comoros and Mascarenes from Madagascar [18,26]. However, only one recent colonisation event is suggested from Africa; the secondary colonization of the species *B. anynana *of Comoros **[256]**.

Sulawesi has been colonised at least twice independently early in the evolution of Mycalesina (between ca. 24-19 mya), by the ancestors of the *Lohora *clade and of *M. itys*. Interestingly, while *Lohora *has radiated into 19 species, the ancestor of *M. itys *seems not to have undergone *in situ *speciation within the island (*M. itys *is the only *Mycalesis *species endemic to Sulawesi).

The endemic Australasian fauna share a common ancestor that was derived from the Oriental region. This ancestor diversified rapidly in New Guinea, with the first splits occurring ca. 15 mya. The endemics in the Solomon Islands (*biliki-splendens-richardi-interrupta-sara*) descend from an ancestor derived from New Guinea in the Late Miocene. *M. terminus *and *M. sirius *(*Mycalesis *II clade) expanded their range into Australia from New Guinea while *M. perseus *(Mycalesis I clade), has colonized Australia and the Solomons between 1 and 2 mya.

### Ecology and evolution

Although some mycalesine faunas, such as that now represented as Clade I of SE Asia, tend to be rather brownish or homogeneous in colour pattern, this is not the case everywhere. The endemics in the Australasian region and the *Lohora *clade of Sulawesi include more colourful members, and may even be mimetic; in the case of *Mycalesis drusillodes *so spectacularly that they had been described as different species, the male mimicking a *Tellervo *and the female a *Taenaris *[13,14]. The Malagasy fauna, in particular, is exceptionally diverse in wing pattern and wing shape. There is also evidence for mimicry between different clades of *Heteropsis *in Madagascar and between *Heteropsis *(*Masoura*) *masoura *(in this case sexually monomorphic) and the aposematic pierid *Mylothris phileris *[7]. Dead leaf mimicry is also evident in the Malagasy *Heteropsis drepana*. The significance of these regional differences in colour pattern and form in different faunas perhaps relates to aspects of the environment, notably differing suites of predators [69], but there is to date no experimental evidence of their role. In Madagascar, mycalesines occurring in open areas or on margins of forests are markedly more orange rather than brown [7].

This remarkable inter-specific diversity in wing patterns may also have been mediated, at least partially, by sexual selection. It has been shown experimentally that eyespots on the dorsal surface of *B. anynana *are used by females to choose mates [47,48]. Pronounced sexual dimorphism occurs in some African *Bicyclus*, and especially within the *Heteropsis *subgenus *Henotesia *in Madagascar, another case extreme enough that females had not been been matched to males, neither had different seasonal stages of males been correctly synonymised (but see [7,70]). Wing patterns are known to have sex-specific evolutionary rates within *Bicyclus *[50]. All these lines of evidence point towards sexual selection as an additional mediator of morphological diversification. Results in [50] also suggested that mate signalling selects for varying dorsal wing patterns while ventral characters are selected upon by predation pressure. Thus, a complex interaction of differential selective forces along with the heterogeneity of environments inhabited by these species seems to have driven the morphological diversification of wing patterns in this group, and much needs to be learnt about the systems.

The most striking aspect of diversification that may be related to speciation in mycalesine butterflies is in the scent organs, which are exceptionally diverse, including male androconia on forewing underside, hindwing upperside, abdominal underside and upperside, specialized wing veins, and specialized polished areas that may have protective function [7]. These androconia are known to release male sex pheromones during courtship, which are crucial determinants of reproductive isolation between species [71,72]. Even to humans, mycalesine scent brushes differ in odour [7]. A denser sampling is needed to test whether the exceptional diversification of androconia is related to elevated levels of speciation in this group. Our phylogeny provides a starting point to identify groups that can be used for further comparative analyses that seek to understand the underlying processes that might have generated these diverse characters.

From a biogeographic perspective, the moderate to weak dispersive powers of mycalesine butterflies explains some of their diversity, because poor dispersive power is expected to be conducive towards higher rates of allopatric speciation [73]. Dispersal into a novel area is more likely to result in allopatric speciation compared to a group with high vagility that can maintain regular geneflow [73]. Vicariance events are also more likely to result in speciation when the ancestor in question has weak dispersive powers. For instance, the aridification of central India during the Pliocene may have resulted in vicariant events that left descendant sister species pairs disjunct in South-West India and North-East India [74]. Unfortunately, we are unable to test this hypothesis with our limited taxon sampling.

The radiation of Satyrini (Satyrinae) butterflies, to which Mycalesina belongs, is thought to have closely followed the diversification of C_4 _grasses during the Oligocene [33]. Host-plant mediated speciation can occur due to episodes of host-expansion and specialization during the history of the group [75,76]. The degree of specialization of satyrines on their grass host plants is unknown, but *Mycalesis *is one satyrine group where host-plant preference hierarchy and specialization has been reported [77]. A rigorous test of the hypothesis that mycalesines radiated by co-evolving with their grass hostplants is, however, hindered by the lack of reliable host plant records for most mycalesines.

We surmise that a combination of several factors - androconial diversification, sexual selection on wing patterns, moderate dispersive powers, and perhaps also co-evolution with grasses - has resulted in the high diversity of species in the group. In summary, this group of butterflies presents an exciting opportunity to understand patterns and processes of diversification in insects, especially in unravelling the complex interactions among various selective forces and developmental aspects of wing patterns. The phylogeny encompassing the entire radiation and nomenclatural rationalisation at a generic level presented here is the first step that will eventually permit large-scale analyses to explore specific hypotheses within a comparative framework.

## Conclusions

Our phylogenetic hypothesis based on three genes indicates that Mycalesina radiated rapidly during the Oligocene-Miocene boundary. Their origin is as yet unclear, and may be either in Asia or Africa, but they have undergone dispersals between the two regions. Our topology implies that Madagascar was colonized at least twice, resulting in independent radiations, but it is not yet certain if its role as a source area was limited to colonisation of neighbouring islands. More clearly, the Australasian mycalesine fauna have radiated following a single dispersal event to the region. We propose a radically new classification of the group and discuss factors likely to have played a key role in their diversification. Our phylogeny paves the way for exciting comparative studies that will help us understand the process of diversification of rapid radiations.

## Methods

### Data collection

Specimens of 42 species of *Mycalesis*, 28 of *Heteropsis*, four of *Lohora *and two of *Nirvanopsis *were collected either by the authors or collaborators at different times between 2001 and 2008. 13 widespread species were represented by more than one sample. DNA was preserved either through dessication or by immersing two legs in alcohol. Once the samples reached the lab, DNA was extracted from two legs using the DNEasy extraction kit (QIAgen). DNA was amplified from three gene regions - 1450 bp (base pairs) of COI (cytochrome oxidase subunit I), a mitochondrial gene, and two nuclear genes - EF-1α (Elongation Factor 1 alpha; 1240 bp) and *wingless *(400 bp). The gene trio has been successful in resolving relationships of species within a genus in several nymphalid studies [65,78-81].

Readers are referred to [82] for a list of primer sequences used here. COI was amplified using the primer pairs LCO-HCO and Jerry-Pat. Three primer pairs were used for EF-1α - Starsky-Luke, Cho-Verdi and EF51.9-EFrcM4, while LepWing1 and LepWing2 or Wingnut 1A (5'-GAA ATG CGN CAR GAR TGY AA-3') and Wingnut-3 (5'-ACY TCR CAR CAC CAR TGR AA-3') were used for *wingless*. The PCR protocol used for Starsky-Luke was as follows -95°C for 7 min, 40 cycles of 95°C for 30 s, 55°C for 30 s and 72°C for 1 min followed by a final extension period of 72°C for 10 min. For Wingnut 1A-Wingnut 3, conditions were 80°C for 1 min, 40 cycles of 94°C for 1 min, 46-52°C for 2 min and 72°C for 1-2 min followed by a final extension period of 72°C for 10 min. For the remaining five primer pairs, we used the following protocol: 95°C for 7 min, 40 cycles of 95°C for 30 s, 50°C for 30 s and 72°C for 1 min followed by a final extension period of 72°C for 10 min. Successfully amplified PCR products were sequenced with a Beckmann-Coulter CEQ8000 automated sequencer. The resulting chromatograms were visualized in BioEdit v7.0.5.3 [83] and aligned by eye. We also included sequence data from the genera *Bicyclus*, *Heteropsis *and *Hallelesis *that were available on Genbank. Outgroup data were taken from [32]. Additional file 5 lists the samples used in this study with their collection localities and Genbank accession numbers for respective sequences.

### Phylogenetic inference

The combined dataset was analyzed under the maximum parsimony (MP) criterion in TNT v 1.1 [84]. Heuristic searches including traditional TBR branch swapping procedures and 'New Technology' searches were performed on 1000 random addition replicates. Support for respective clades was estimated using bootstrap values calculated from 1000 pseudo-replicates with 10 replicates each. Maximum likelihood (ML) analyses were performed in RAxML III [85] with default heuristic search algorithms. The GTR+G model, which was chosen by jModelTest [86] under the Akaike Information Criterion, was imposed on the three gene partitions independently with the gamma parameter estimated in 4 discrete rate categories. Bootstrap values were calculated from 1000 pseudo-replicates. Since some samples did not have a complete three-gene dataset (Additional file 6), ML analyses were performed on individual gene datasets excluding samples missing data for respective genes. Individual gene analyses also allow us to assess nodal support from each gene.

We performed Shimodaira-Hasegawa tests [87] to test whether specific topologies resulting from the above analyses were significantly better than competing topologies. The program MacClade [88] was used to construct two constraint trees; in the first tree each genus were forced to be monophyletic and in the second tree only species in *Mycalesis *clades I and II (see results) were placed in a monophyletic group, while the position of other species remained unconstrained. These constraint trees were used in PAUP* [89] to derive a "genus monophyly" tree and "*Mycalesis *monophyly" tree through a likelihood heuristic search. To examine support for the above hypotheses the likelihood scores of these trees were compared with the unconstrained ML tree using the one-tailed Shimodaira-Hasegawa log-likelihood test as implemented in PAUP*, using the re-sampling estimated log-likelihood (RELL) technique approximation with 10,000 bootstrap replications. These analyses were implemented on the combined dataset.

We also conducted a 'rogue' analysis to test whether there was one or more 'rogue' clades that was leading to the observed low basal support values. Each of the six 'stable' clades identified in the Results section was successively removed and the datasets analysed in RaxML with bootstrapping to test whether deletion of one of these clades resulted in stronger support.

### Estimate of divergence times

We used the software BEAST v 1.4.8 [90] for Bayesian inference (BI) of phylogenetic relationships and divergence times simultaneously. The analysis was carried out without outgroups since BEAST does not rely on outgroups to root the tree; instead it uses a relaxed molecular clock. The "treeModel.RootHeight" prior (i.e., the age at the root of the tree) was set to a normal distribution with a mean of 27 million years and a standard deviation of 3. This date was taken from [64] which studied the divergence times within Nymphalidae based on a 10-gene dataset from >400 genera (including *Mycalesis*, *Bicyclus, Hallelesis *and *Heteropsis*), with both minimum and maximum calibration points. The dataset was partitioned into nuclear (EF-1α and wingless combined) and mitochondrial (COI) genes, with parameter values estimated independently for each partition. The GTR+G model was imposed with a relaxed clock where branch lengths were allowed to vary according to an uncorrelated lognormal distribution [91]. The tree prior was set to the Birth-Death process, while all other priors were left to their defaults in BEAST. The analysis was run twice for 10,000,000 generations of MCMC analyses in BEAST and the chains were sampled at every 1,000 generations, yielding a total of 10,000 samples for each run. Whether the parameter estimates and tree topology were at equilibrium was determined by using the program Tracer [91]. The first 1,000,000 generations (or 1000 trees) were discarded as burn-in.

## Authors' contributions

UK and NW conceptualized and coordinated the study, with the former doing a major part of the lab work. DCL, PK, ET and NW provided sequence data while CJM collected most of the samples from the Australasian region used here. The manuscript was drafted by UK with active participation from the rest of the authors. All authors read and approved the final manuscript.

## Supplementary Material

Additional files 1 **Appendix 1**. List of genera under which Mycalesis was divided under by Moore (1880), and the species groupings of Evans (1932) and Aoki et al (1982). Click here for file

Additional file 2 **Appendix 2**. Maximum Likelihood topology recovered from the RAxML analysis of the COI dataset. Numbers indicate bootstrap support for the nodes to the right. Click here for file

Additional file 3 **Appendix 3**. Maximum Likelihood topology recovered from the RAxML analysis of the EF-1α dataset. Numbers indicate bootstrap support for the nodes to the right. Click here for file

Additional file 4 **Appendix 4**. Maximum Likelihood topology recovered from the RAxML analysis of the *wingless *dataset. Numbers indicate bootstrap support for the nodes to the right. Click here for file

Additional file 5 **Appendix 5**. Maximum Likelihood topologies recovered in RAxML analyses where each of the six stable clades were successively removed from the dataset. Numbers indicate bootstrap support for the nodes to the right. a) minus *Bicyclus*, b) minus clade 1, c) minus *Mycalesis *clade 2, d) minus *Hallelesis*, e) minus *Heteropsis*, f) minus *Lohora*. Click here for file

Additional file 6 **Appendix 6**. List of taxa used in this study with their Genbank accession numbers. An asterix after the name indicates that *wingless *for that species was sequenced from a different individual of the same species. A double asterisk indicates that the taxon was an outgroup. Collection localities are mentioned for all samples collected for the purpose of this study. Click here for file
