# Supplementary material for: Phylogenetics and biogeography of a spectacular Old World radiation of butterflies: the subtribe Mycalesina (Lepidoptera: Nymphalidae: Satyrini)
Source: BMC Evol Biol. 2010 Jun 10;10:172. doi: 10.1186/1471-2148-10-172 (PMC2898688; doi:10.1186/1471-2148-10-172)
Supplement: Additional files 1 — Appendix 1. List of genera under which Mycalesis was divided under by Moore (1880), and the species groupings of Evans (1932) and Aoki et al (1982). [file 1471-2148-10-172-S1.PDF]

Appendix 1

Moore (1880) divided the Indian and other Indo-Australasian species then known into no less than 22 genera, of which 20 are included in this work (viz: in bold). At the time of Moore's work, the type species *of Mycalesis* was considered to be the West African *Papilio evadne* Cramer, [1779], a *Bicyclus*. It was not until 1937 that Hemming selected the type species as *Papilio francisca* Cramer, a species Moore included in *Gareris*.

The species Moore treated in new genera (according to wing shape and venation and scent organ configurations) unless otherwise mentioned (not here updated for synonymy; taxa sampled by us are in bold) were:

1. ***Virapa*** (***anaxias***, *radza*)
2. ***Gareris*** (*sanatana*, ***francisca***, *perdiccas*, *gopa*)
3. ***Satoa*** **(*maianeas*)**
4. ***Sadarga*** (***gotama***, *madjicosa*, *oculata*, *charaka*)
5. *Dalapa* (*sudra*)
6. ***Suralaya*** (***orseis***)
7. ***Calysisme*** (*drusia*, *justina*, ***mamerta***, ***mineus***, *polydecta*, *igoleta*, *nautilus*, *justinella blasius*, *samba*, *lalassis*, *cepheus*, *lugens*, *zia*, ***perseus***, *indistans*, *ostrea*, ***visala***)
8. ***Jatana*** (***mynois***)
9. *Culapa* (*mnasicles*)
10. *Pachama* (*mestra*)
11. *Indalasa* (*moorei*)
12. ***Samanta*** (***malsara***, *rudis*, *lepcha*, *heri*, *nicotia*)
13. ***Telinga*** (***adolphei***)
14. *Kabanda* (*malsarida*, *khasiana*)
15. ***Martanda*** (***janardana***, *megamede*, ***sangaica***)
16. ***Nissanga*** (***patnia***, *junonia*)
17. ***Mydosama*** (***fuscum***, ***anapita***, *remutia*, ***terminus***, ***flagrans***, ***cacodemon***, *messene* ***mehadeva***, ***asophis***, ***aethiops***, *bazochi*, *ita*, *felderi*, ***itys***, ***sirius***, *zachaeus*, *manipa shiva*, ***phidon***, *caesonia*)
18. *Nebdara* (*tagala*, *bisaya*), *amoena*
19. ***Sevanda*** (***duponcheli***, ***mucia***)
20. ***Lohora*** (*dexamena*, *dinon*, *deianira*)
21. *Nasapa* (*aramis*)
22. *Loesa* (*oroatis*)

"incertae sedis": *nala*, *pandaea*.

Evans (1932) divided Indian *Mycalesis* species into five groups, as follows:

1. Gotama group: ***maianeas***, *adamsoni*, ***anaxias***, *anaxioides*, ***francisca***, ***gotama***, ***orseis***, ***fuscum***, ***anapita***
2. Mineus group: ***perseus***, ***mineus***, *igilia*, ***visala***, ***perseoides***, *subtida*, *mercea*, *khasia*, *rama*, *evansii*, *mystes*, ***adolphei***, *oculus*
3. Oroatis group: *oroatis*
4. Nicotia group: *mnasicles*, ***suaveolens***, *mestra*, *heri*, *nicotia*, ***malsara***, ***mamerta***, *lepcha*, *malsarida*
5. Patnia group: ***patnia***

Talbot, G. (1947; The Fauna of British India, Ceylon and Burma: Volume 2) divided *Mycalesis* species into seven groups:

1. Francisca group: ***Virapa***, ***Gareris***, ***Satoa***, ***Sadarga***, ***Suralaya*** of Moore (1880) and *Samundra* of Moore (1891).
2. Fuscum group: ***Dasyomma*** of Moore.
3. Mineus group: ***Calysisme*** and ***Telinga*** of Moore.
4. Mystes group: *Myrtilus* of de Nicéville.
5. Nicotia group:*Culapa*, *Pachama*, ***Samanta*** and *Kabanda* of Moore.
6. Patnia group: ***Nissanga*** of Moore.
7. Oroatis group: *Loesa* of Moore.

Aiko *et al* (1982) divided the genus into five species groups:

1) 1-species group: ***anaxias**, **francisca**, adamsoni, deficiens,* ***gotama**, **orseis***
2) 2-species group: ***perseus**, subpersa,* ***mineus**, igilia,* ***visala**, **perseoides**, **intermedia**, mercea, rama, igoleta, evansii, oculus*
3) 3-species group: *malsarida, heri, mestra, suaveolens, nicotia, misenus,* ***mamerta**, **malsara**, annamitica, lepcha*
4) 4-species group: ***patnia***
5) 5-species group: *mystes*
