# Supplementary figures and images for: Phylogenetics and biogeography of a spectacular Old World radiation of butterflies: the subtribe Mycalesina (Lepidoptera: Nymphalidae: Satyrini)
Source: BMC Evol Biol. 2010 Jun 10;10:172. doi: 10.1186/1471-2148-10-172 (PMC2898688; doi:10.1186/1471-2148-10-172)

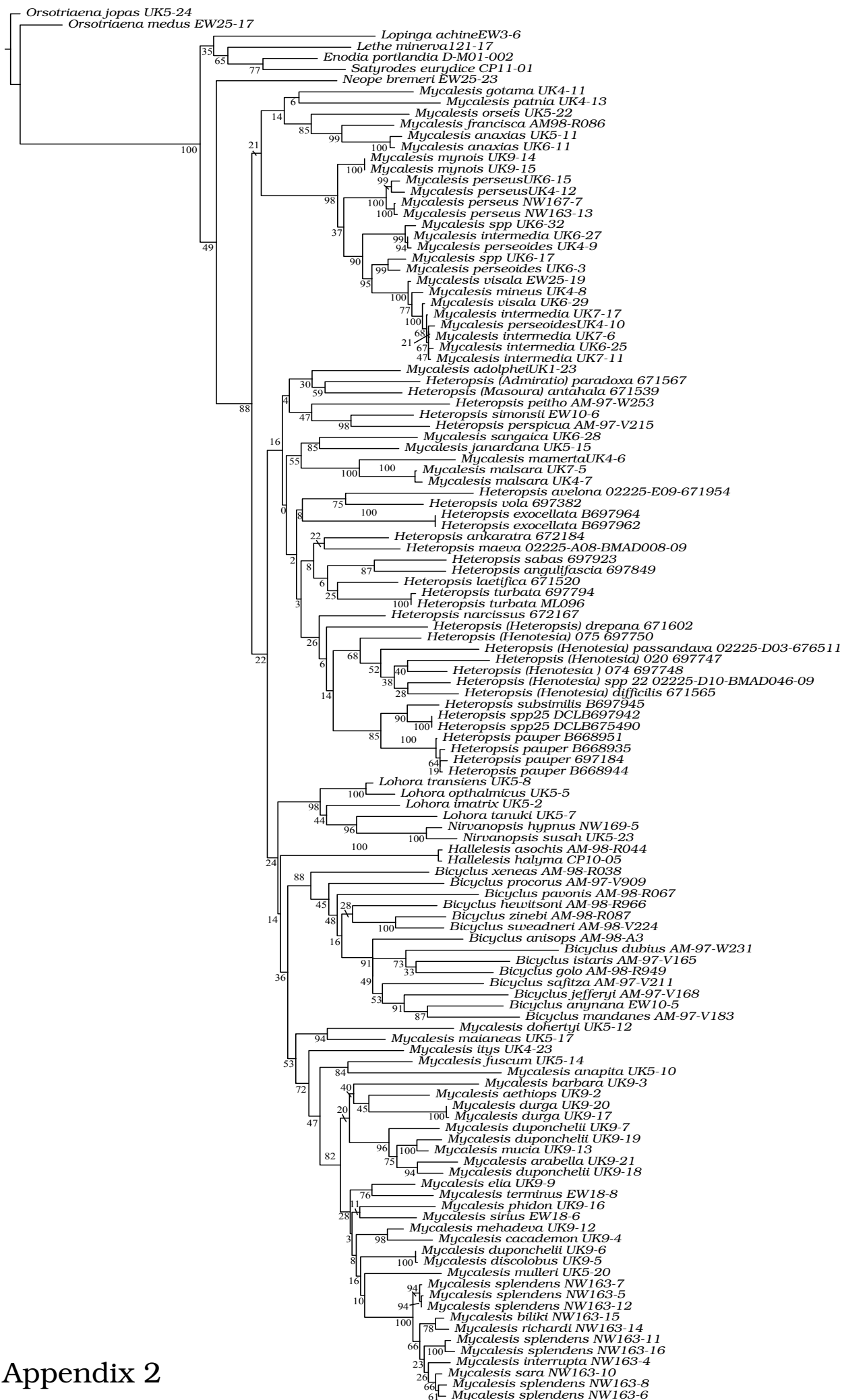

Appendix 2

Supplement: Additional file 2 — Appendix 2. Maximum Likelihood topology recovered from the RAxML analysis of the COI dataset. Numbers indicate bootstrap support for the nodes to the right. [file 1471-2148-10-172-S2.PDF]

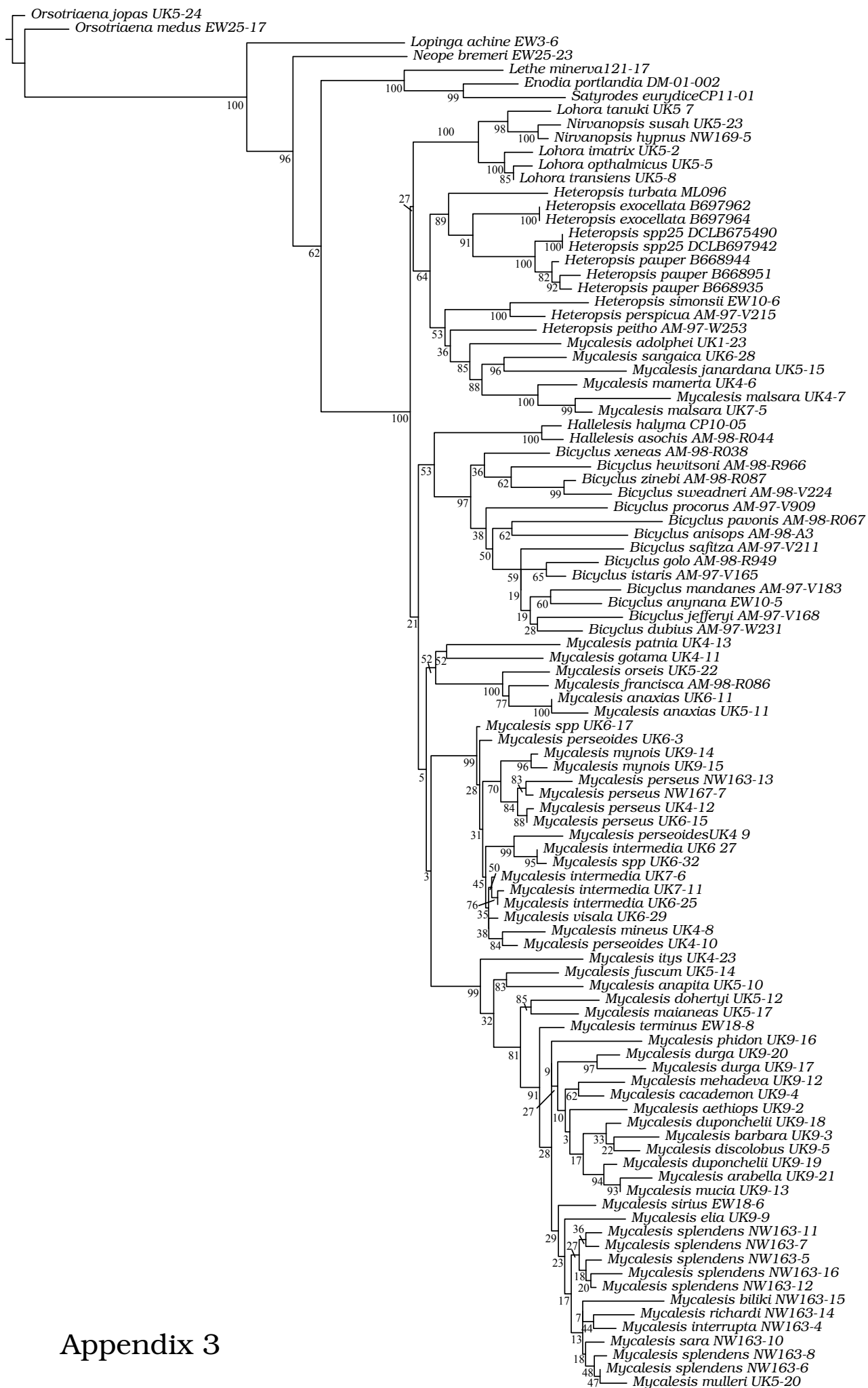

## Appendix 3

Supplement: Additional file 3 — Appendix 3. Maximum Likelihood topology recovered from the RAxML analysis of the EF-1α dataset. Numbers indicate bootstrap support for the nodes to the right. [file 1471-2148-10-172-S3.PDF]

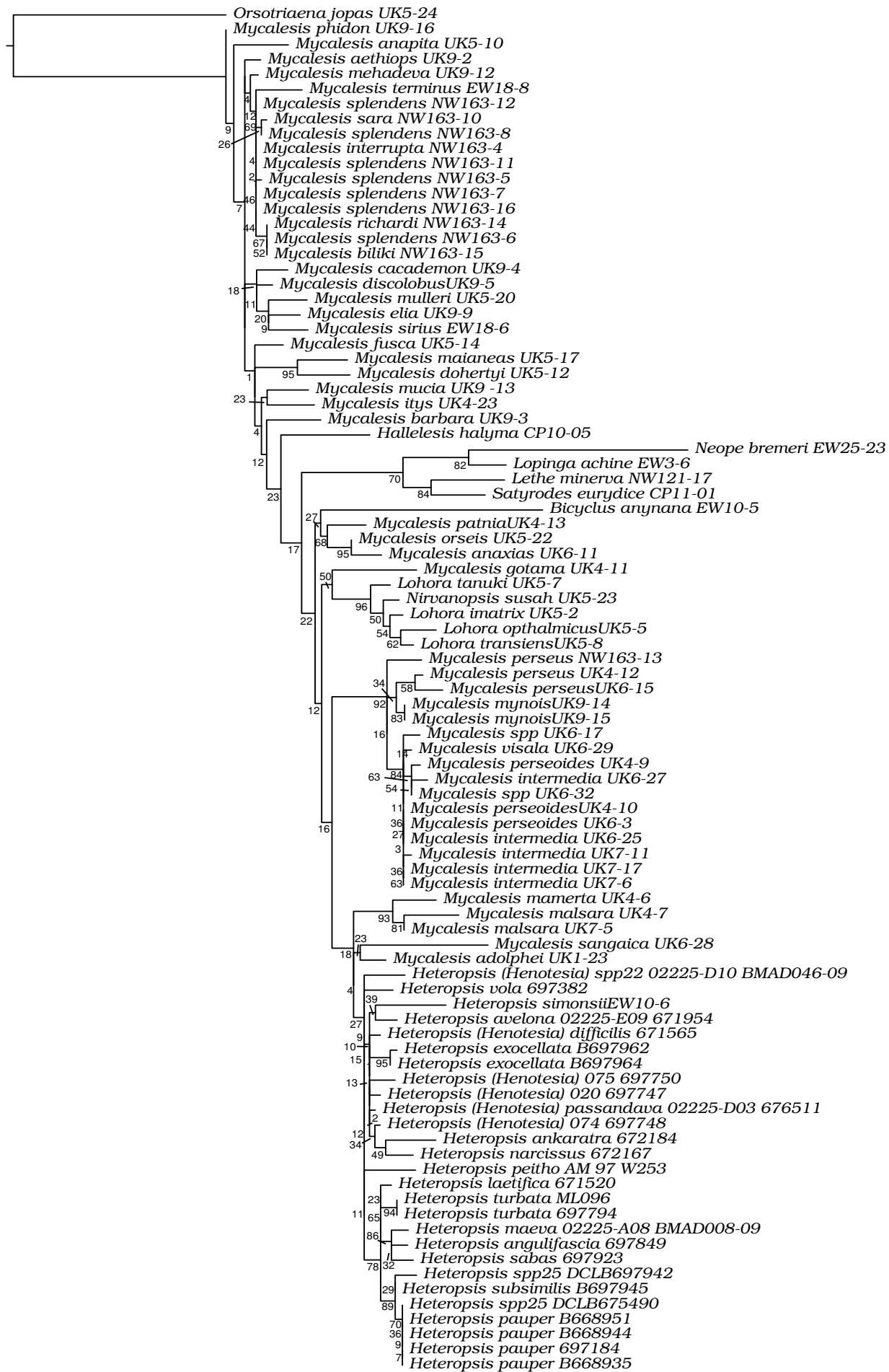

## Appendix 4

Supplement: Additional file 4 — Appendix 4. Maximum Likelihood topology recovered from the RAxML analysis of the wingless dataset. Numbers indicate bootstrap support for the nodes to the right. [file 1471-2148-10-172-S4.PDF]

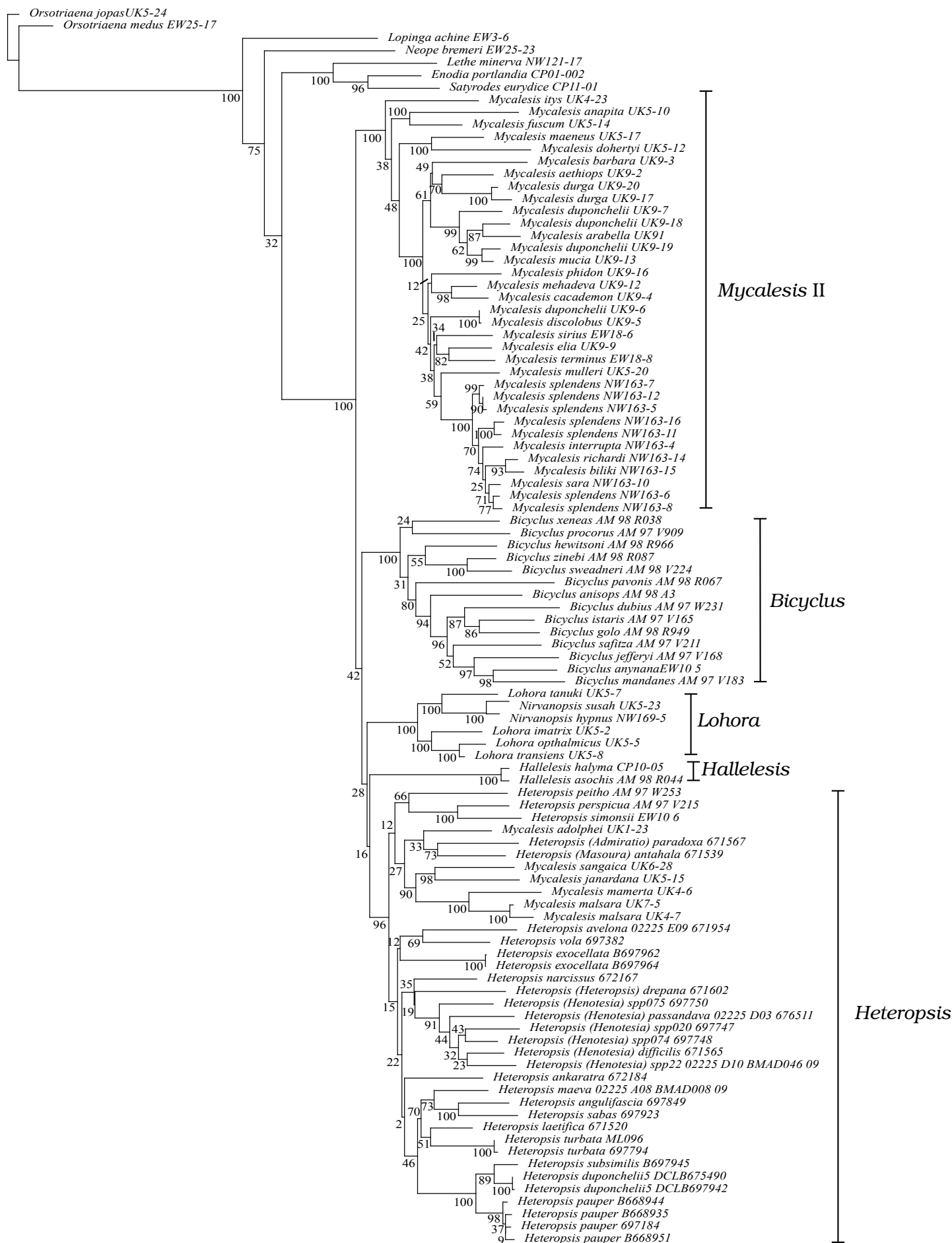

Appendix 5b

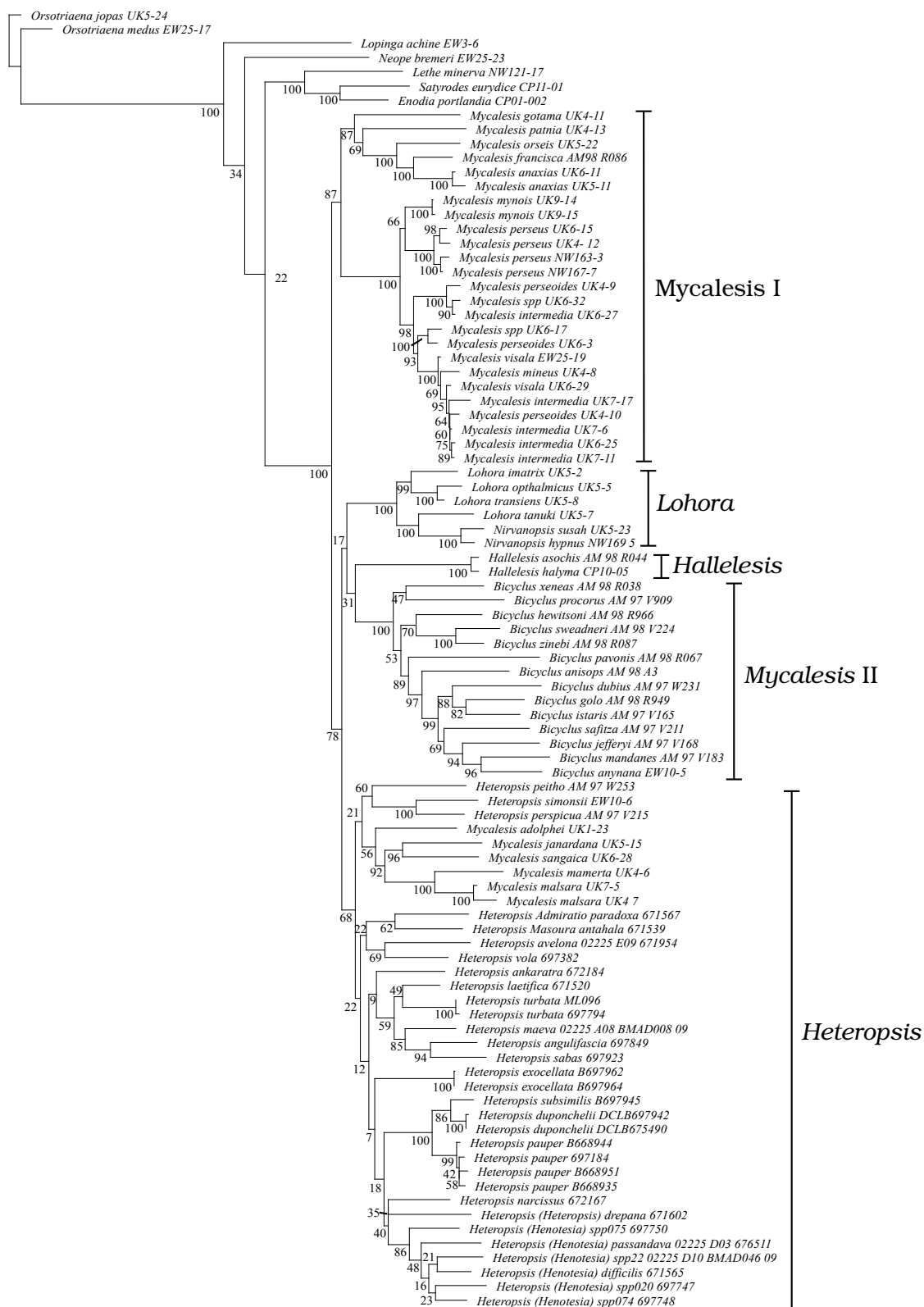

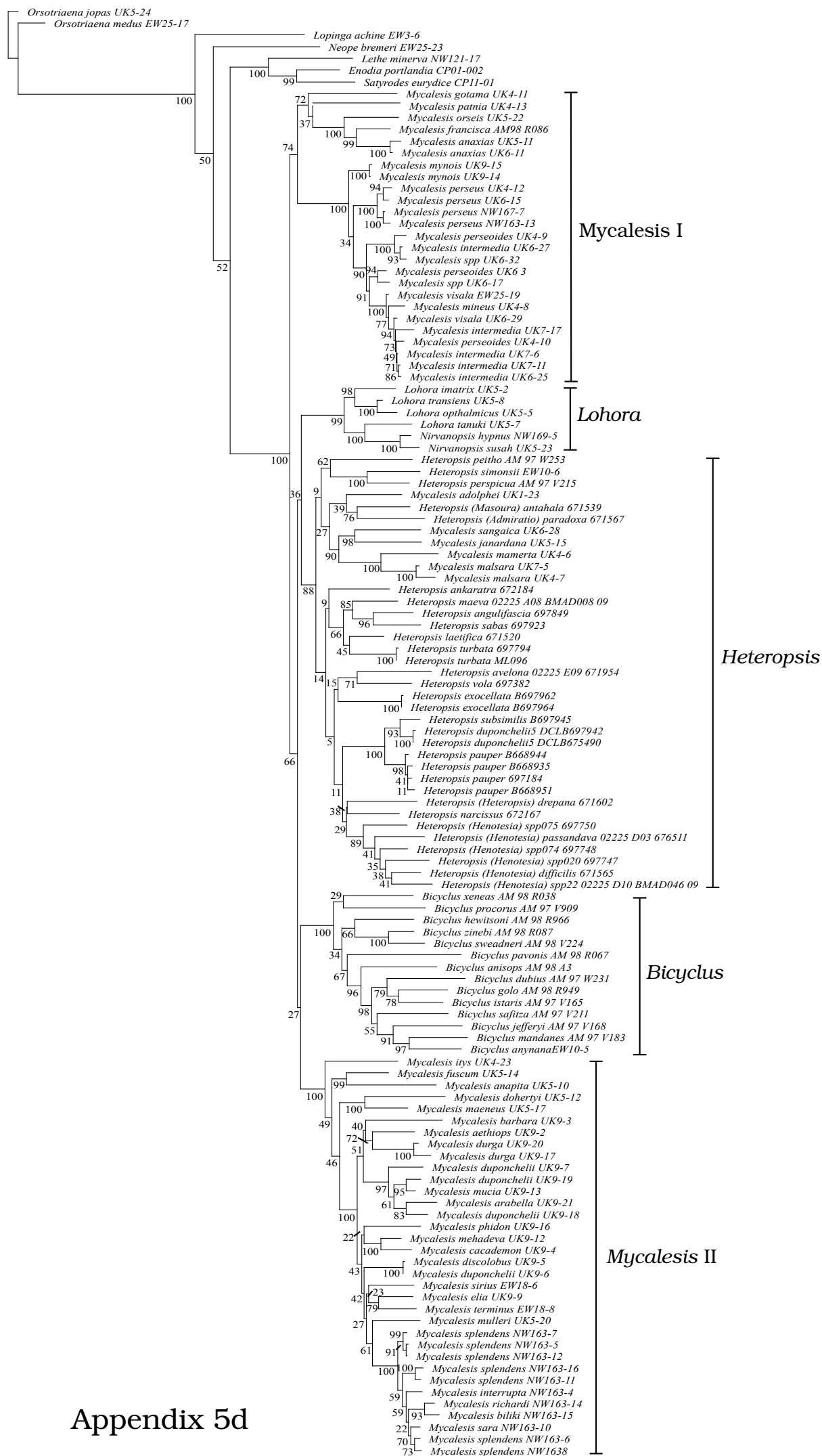

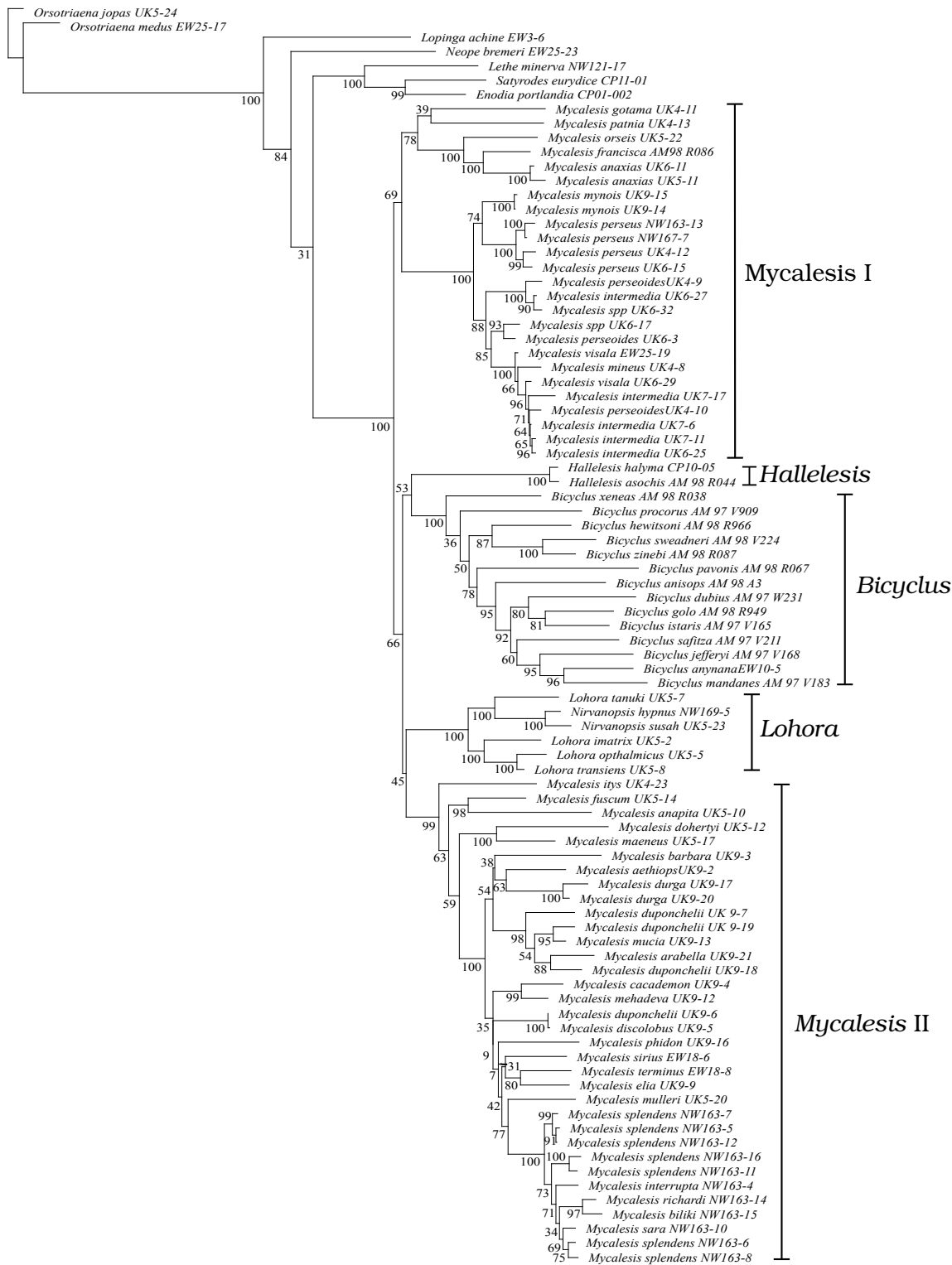

## Appendix 5e

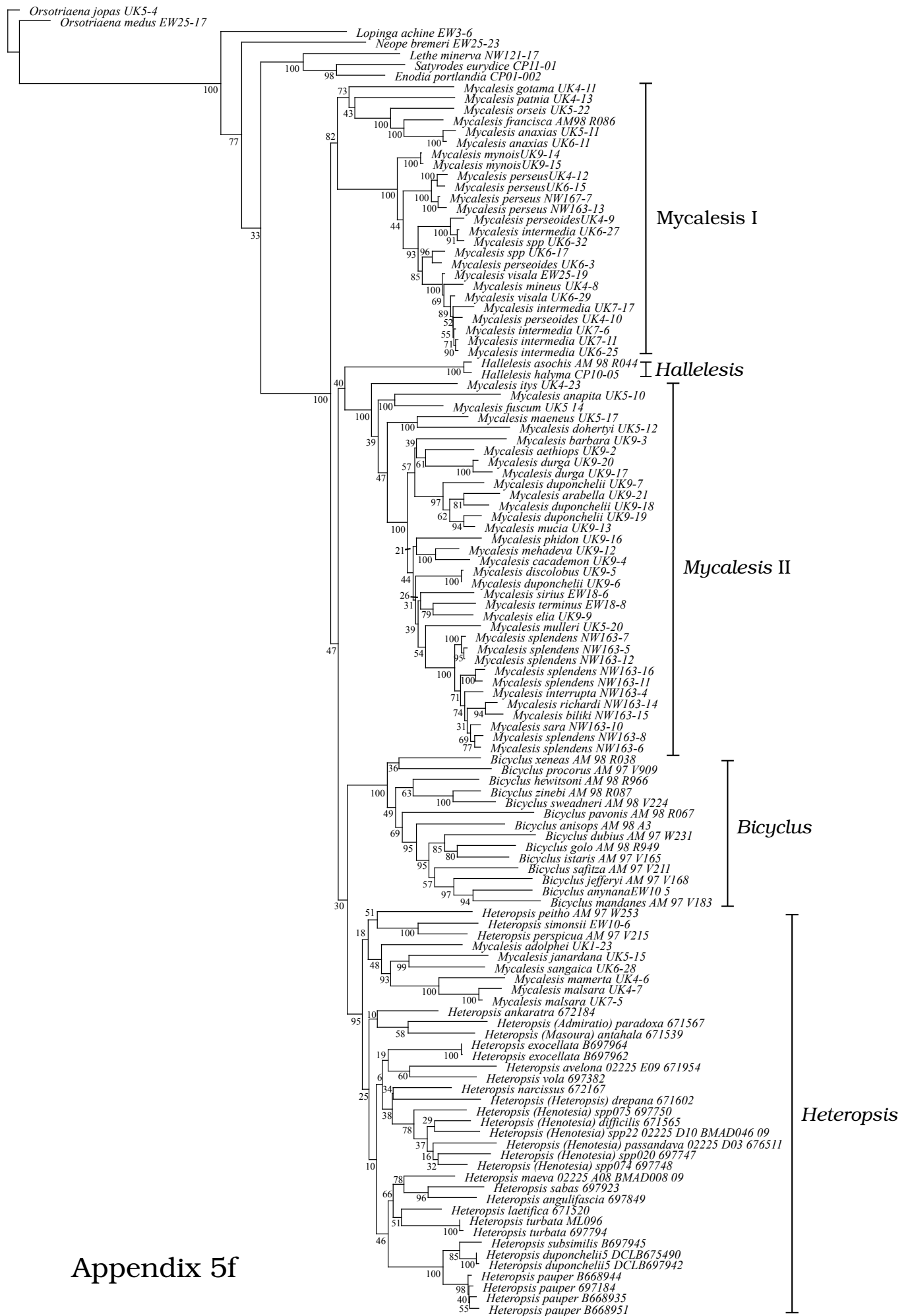

Appendix 5f

Supplement: Additional file 5 — Appendix 5. Maximum Likelihood topologies recovered in RAxML analyses where each of the six stable clades were successively removed from the dataset. Numbers indicate bootstrap support for the nodes to the right. a) minus Bicyclus, b) minus clade 1, c) minus Mycalesis clade 2, d) minus Hallelesis, e) minus Heteropsis, f) minus Lohora. [file 1471-2148-10-172-S5.PDF]
