# Supplementary material for: Phylogenetics and biogeography of a spectacular Old World radiation of butterflies: the subtribe Mycalesina (Lepidoptera: Nymphalidae: Satyrini)
Source: BMC Evol Biol. 2010 Jun 10;10:172. doi: 10.1186/1471-2148-10-172 (PMC2898688; doi:10.1186/1471-2148-10-172)
Supplement: Additional file 6 — Appendix 6. List of taxa used in this study with their Genbank accession numbers. An asterix after the name indicates that wingless for that species was sequenced from a different individual of the same species. A double asterisk indicates that the taxon was an outgroup. Collection localities are mentioned for all samples collected for the purpose of this study. [file 1471-2148-10-172-S6.PDF]

Appendix 6

Voucher ID	Species	Collection locality	COI	EF1al	wingless
AM-98-A3	*Bicyclus anisops*	N/A	HM241421	HM241537	Nil
EW10-5	*Bicyclus anynana*	Harare, Zimbabwe	AY218238	AY218258	AY218276
AM-97-W231	*Bicyclus dubius*	N/A	HM241429	HM241545	Nil
AM-98-R949	*Bicyclus golo*	N/A	HM241432	HM241549	Nil
AM-98-R966	*Bicyclus hewitsoni*	N/A	HM241435	HM241552	Nil
AM-97-V165	*Bicyclus istaris*	N/A	HM241441	HM241558	Nil
AM-97-V168	*Bicyclus jefferyi*	N/A	HM241443	HM241560	Nil
AM-97-V183	*Bicyclus mandanes*	N/A	HM241446	HM241563	Nil
AM-98-R067	*Bicyclus pavonis*	N/A	HM241453	HM241570	Nil
AM-97-V909	*Bicyclus procorus*	N/A	HM241455	HM241572	Nil
AM-97-V211	*Bicyclus safitza*	N/A	HM241458	HM241575	Nil
AM-98-V224	*Bicyclus sweadneri*	N/A	HM241467	HM241584	Nil
AM-98-R038	*Bicyclus xeneas*	N/A	HM241474	HM241591	Nil
AM-98-R087	*Bicyclus zinebi*	N/A	HM241476	HM241593	Nil
AM-98-R044	*Hallelesis asochis*	N/A	HM241434	HM241551	Nil
CP10-05	*Hallelesis halyma*	Ghana	DQ338763	DQ338903	DQ338630
ANG8M	*Heteropsis angulifascia*	Madagascar	Nil	Nil	HM240772
697849	*Heteropsis angulifascia**	Madagascar	HM240633	Nil	Nil
ANK1M	*Heteropsis ankaratra*	Madagascar	Nil	Nil	HM240764
672184	*Heteropsis ankaratra**	Madagascar	HM240625	Nil	Nil
9666	*Heteropsis avelona*	Madagascar	Nil	Nil	x
02225-E09-671954	*Heteropsis avelona**	Madagascar	HM240624	Nil	Nil
B697962	*Heteropsis exocellata*	Madagascar	FJ666697	FJ666598	FJ666721
B697964	*Heteropsis exocellata*	Madagascar	FJ666699	FJ666597	FJ666720
671520	*Heteropsis laetifica**	Madagascar	HM240627	Nil	Nil
9690	*Heteropsis laetifica*	Madagascar	Nil	Nil	HM240766
02225-A08-BMAD008-09	*Heteropsis maeva**	Madagascar	HM240626	Nil	Nil
MAE7M	*Heteropsis maeva*	Madagascar	Nil	Nil	HM240765
672167	*Heteropsis narcissus**	Madagascar	HM240632	Nil	Nil
NAR2M	*Heteropsis narcissus*	Madagascar	Nil	Nil	HM240771
B668951	*Heteropsis pauper*	Madagascar	FJ666635	FJ666600	FJ666723
B668944	*Heteropsis pauper*	Madagascar	FJ666612	FJ666610	FJ666733
B668935	*Heteropsis pauper*	Madagascar	FJ666631	FJ666606	FJ666729
697184	*Heteropsis pauper**	Madagascar	HM240631	Nil	Nil
7A1F	*Heteropsis pauper*	Madagascar	Nil	Nil	HM240770
AM-97-W253	*Heteropsis peitho*	N/A	HM241436	HM241553	Nil
AM-97-V215	*Heteropsis perspicua*	N/A	HM241437	HM241554	Nil
697923	*Heteropsis sabas**	Madagascar	HM240629	Nil	Nil
9682	*Heteropsis sabas*	Madagascar	Nil	Nil	HM240768
EW10-6	*Heteropsis simonsii*	Harare, Zimbabwe	DQ338764	DQ338904	DQ338631
DCLB675490	*Heteropsis spp25*	Madagascar	FJ666685	FJ666601	FJ666724
DCLB697942	*Heteropsis spp25*	Madagascar	FJ666683	FJ666608	FJ666731
B697945	*Heteropsis subsimilis*	Madagascar	FJ666676	FJ666599	FJ666722
697794	*Heteropsis turbata**	Madagascar	HM240628	Nil	Nil
8C06M	*Heteropsis turbata*	Madagascar	Nil	Nil	HM240767
ML096	*Heteropsis turbata*	Madagascar	FJ666707	FJ666603	FJ666726
697382	*Heteropsis vola**	Madagascar	HM240630	Nil	Nil
VOL1F	*Heteropsis vola*	Madagascar	Nil	Nil	HM240769
671567	*H. (Admiratio) paradoxa*	Madagascar	HM240616	Nil	Nil

671565	*H. (Henotesia) difficilis**	Madagascar	HM240621	Nil	Nil
10c10M	*H. (Heteropsis) difficilis*	Madagascar	Nil	Nil	HM240760
02225-D03-676511	*H. (Heteropsis) passandava**	Madagascar	HM240623	Nil	Nil
HO2A3	*H. (Heteropsis) passandava*	Madagascar	Nil	Nil	HM240762
697747	*H. (Heteropsis) spp020**	Madagascar	HM240620	Nil	Nil
9661M	*H. (Heteropsis) spp020*	Madagascar	Nil	Nil	HM240759
697748	*H. (Heteropsis) spp074**	Madagascar	HM240622	Nil	Nil
9683	*H. (Heteropsis) spp074*	Madagascar	Nil	Nil	HM240761
697750	*H. (Heteropsis) spp075**	Madagascar	HM240619	Nil	Nil
9670	*H. (Heteropsis) spp075*	Madagascar	Nil	Nil	HM240758
02225-D10-BMAD046-09	*H. (Heteropsis) spp22**	Madagascar	HM240634	Nil	Nil
22D4M	*H. (Heteropsis) spp22*	Madagascar	Nil	Nil	HM240773
671602	*H. Heteropsis) drepana*	Madagascar	HM240618	Nil	Nil
671539	*H. (Masoura) antahala*	Madagascar	HM240617	Nil	Nil
UK5-2	*Lohora imatrix*	Central Sulawesi, Indonesia	HM240578	HM240666	HM240727
UK5-5	*Lohora opthalmicus*	South Sulawesi, Indonesia	HM240582	HM240670	HM240731
UK5-7	*Lohora tanuki*	Central Sulawesi, Indonesia	HM240583	HM240671	HM240732
UK5-8	*Lohora transiens*	Central Sulawesi, Indonesia	HM240584	HM240672	HM240733
UK1-23	*Mycalesis adolphei*	Coorg, Karnataka, India	HM240564	HM240650	HM240714
UK9-2	*Mycalesis aethiops*	Morobe Province, Papua New Guinea	HM240606	HM240693	HM240752
UK5-10	*Mycalesis anapita*	Kalimantan, Indonesia	HM240572	HM240660	HM240723
UK5-11	*Mycalesis anaxias*	Souhern Thailand	HM240573	HM240661	Nil
UK6-11	*Mycalesis anaxias*	Chalsenouk, Luang Nam Tha, Laos	HM240585	HM240673	HM240734
UK9-21	*Mycalesis arabella*	Waigeo, Irian Jaya, Indonesia	HM240608	HM240695	Nil
UK9-3	*Mycalesis barbara*	Hidden Valley, Irian Jaya, Indonesia	HM240609	HM240696	HM240753
NW163-15	*Mycalesis biliki*	Malaita, Solomon Islands	HM240553	HM240641	HM240707
UK9-4	*Mycalesis cacademon*	Morobe Province, Papua New Guinea	HM240610	HM240697	HM240754
UK9-5	*Mycalesis discolobus*	Hidden Valley, Irian Jaya, Indonesia	HM240611	HM240698	HM240755
UK5-12	*Mycalesis dohertyi*	Kalimantan, Indonesia	HM240574	HM240662	HM240724
UK9-18	*Mycalesis duponchelii*	Waigeo, Irian Jaya, Indonesia	HM240604	HM240691	Nil
UK9-19	*Mycalesis duponchelii*	Waigeo, Irian Jaya, Indonesia	HM240605	HM240692	Nil
UK9-6	*Mycalesis duponchelii*	Yapen Island, Irian Jaya, Indonesia	HM240612	Nil	Nil
UK9-7	*Mycalesis duponchelii*	Morobe Province, Papua New Guinea	HM240613	Nil	Nil
UK9-17	*Mycalesis durga*	Yapen Island, Irian Jaya, Indonesia	HM240603	HM240690	Nil
UK9-20	*Mycalesis durga*	Yapen Island, Irian Jaya, Indonesia	HM240607	HM240694	Nil
UK9-9	*Mycalesis elia*	Morobe Province, Papua New Guinea	HM240614	HM240699	HM240756
AM-98-R086	*Mycalesis francisca*	N/A	HM241450	HM241567	Nil
UK5-14	*Mycalesis fuscum*	Kalimantan, Indonesia	HM240575	HM240663	HM240725
UK4-11	*Mycalesis gotama*	Shi Lin, Yun Nan, China	HM240564	HM240652	HM240716
UK6-25	*Mycalesis intermedia*	Pak Beng, Oudomxai, Laos	HM240588	HM240676	HM240737
UK6-27	*Mycalesis intermedia*	Nong Khiaw, Luang Prabhang,Laos	HM240589	HM240677	HM240738
UK7-11	*Mycalesis intermedia*	Ban Donexay, Luang Nam Tha, Laos	HM240594	HM240682	HM240743
UK7-17	*Mycalesis intermedia*	Vang Vieng, Vientiane, Laos	HM240595	Nil	HM240744
UK7-6	*Mycalesis intermedia*	Nong Khiaw, Luang Prabhang, Laos	HM240597	HM240684	HM240746
NW163-4	*Mycalesis interrupta*	Kolombangara, Solomon Islands	HM240555	HM240643	HM240709
UK4-23	*Mycalesis itys*	South Sulawesi, Indonesia	HM240567	HM240655	HM240719
UK5-15	*Mycalesis janardana*	South Sulawesi, Indonesia	HM240576	HM240664	Nil
UK5-17	*Mycalesis maeneus*	Kalimantan, Indonesia	HM240577	HM240665	HM240726
UK4-7	*Mycalesis malsara*	Cuc Phuong, Vietnam	HM240569	HM240657	HM240721
UK7-5	*Mycalesis malsara*	Nong Khiaw, Luang Prabhang, Laos	HM240596	HM240683	HM240745
UK4-6	*Mycalesis mamerta*	Chong Zuo, Guang Shi, China	HM240568	HM240656	HM240720
UK9-12	*Mycalesis mehadeva*	Yapen Island, Irian Jaya, Indonesia	HM240598	HM240685	HM240747
UK4-8	*Mycalesis mineus*	Shek Kong, Hong Kong	HM240570	HM240658	Nil

UK9-13	*Mycalesis mucia*	Morobe Province, Papua New Guinea	HM240599	HM240686	HM240748
UK5-20	*Mycalesis mulleri*	New Ireland, Papua New Guinea	HM240579	HM240667	HM240728
UK9-14	*Mycalesis mynois*	West Timor, Indonesia	HM240600	HM240687	HM240749
UK9-15	*Mycalesis mynois*	West Timor, Indonesia	HM240601	HM240688	HM240750
UK5-22	*Mycalesis orseis*	Kalimantan, Indonesia	HM240580	HM240668	HM240729
UK4-13	*Mycalesis patnia*	Coorg, Karnataka, India	HM240566	HM240654	HM240718
UK4-10	*Mycalesis perseoides*	Cuc Phuong, Vietnam	HM240563	HM240651	HM240715
UK4-9	*Mycalesis perseoides*	Cuc Phuong, Vietnam	HM240571	HM240659	HM240722
UK6-3	*Mycalesis perseoides*	Luang Nam Tha, Laos	HM240592	HM240680	HM240741
NW163-13	*Mycalesis perseus*	Kolombangara, Solomon Islands	HM240551	HM240639	HM240705
NW167-7	*Mycalesis perseus*	NT: Gurrumuru Outstation, Australia	HM240560	HM240648	Nil
UK4-12	*Mycalesis perseus*	Coorg, Karnataka, India	HM240565	HM240653	HM240717
UK6-15	*Mycalesis perseus*	Ban Nam Lai, Luang Nam Tha, Laos	HM240586	HM240674	HM240735
UK9-16	*Mycalesis phidon*	Morobe Province, Papua New Guinea	HM240602	HM240689	HM240751
NW163-14	*Mycalesis richardi*	Guadalcanal, Solomon Islands	HM240552	HM240640	HM240706
UK6-28	*Mycalesis sangaica*	Ban Nam Thoung, Bokeo, Laos	HM240590	HM240678	HM240739
NW163-10	*Mycalesis sara*	San Cristobal, Solomon Islands	HM240548	HM240636	HM240702
EW18-6	*Mycalesis sirius*	Cairns, Australia	HM240546	HM240635	HM240701
NW163-11	*Mycalesis splendens*	Russell Group, Pavuvu, Solomon Islands	HM240549	HM240637	HM240703
NW163-12	*Mycalesis splendens*	Shortland group, Maleai, Solomon Islands	HM240550	HM240638	HM240704
NW163-16	*Mycalesis splendens*	Guadalcanal, Solomon Islands	HM240554	HM240642	HM240708
NW163-5	*Mycalesis splendens*	Shortland group, Fauro, Solomon Islands	HM240556	HM240644	HM240710
NW163-6	*Mycalesis splendens*	Malaita, Solomon Islands	HM240557	HM240645	HM240711
NW163-7	*Mycalesis splendens*	Mono Island, Solomon Islands	HM240558	HM240646	HM240712
NW163-8	*Mycalesis splendens*	Ulawa, Solomon Islands	HM240559	HM240647	HM240713
UK6-17	*Mycalesis spp*	Chalsenouk, Luang Nam Tha, Laos	HM240587	HM240675	HM240736
UK6-32	*Mycalesis spp*	Pak Beng, Oudomxai, Laos	HM240593	HM240681	HM240742
EW18-8	*Mycalesis terminus*	Cairns, Australia	DQ338765	DQ338905	DQ338632
EW25-19	*Mycalesis visala*	Sylhet Div. Lowacherra Forest, Bangladesh	HM240547	Nil	Nil
UK6-29	*Mycalesis visala*	Ban Donexay, Luang Nam Tha, Laos	HM240591	HM240679	HM240740
NW169-5	*Nirvanopsis hypnus*	Sareong, Central Sulawesi, Indonesia	HM240561	HM240649	Nil
UK5-23	*Nirvanopsis susah*	Taliabu Island, Indonesia	HM240581	HM240669	HM240730
DM-01-002**	*Enodia portlandia*	USA	AY508536	AY509062	Nil
NW121-17**	*Lethe minerva*	Bali, Indonesia	DQ338768	DQ338909	DQ338616
EW3-6**	*Lopinga achine*	Sweden	DQ338769	DQ338910	DQ338617
EW25-23**	*Neope bremeri*	Wutai Shiang, Pingtung County , Taiwan	DQ338770	DQ338911	DQ338618
UK5-24**	*Orsotriaena jopas*	Central Sulawesi, Indonesia	HM240615	HM240700	HM240757
EW25-17**	*Orsotriaena medus*	Sylhet Div. Lowacherra Forest, Bangladesh	DQ338766	DQ338906	DQ338633
CP11-01**	*Satyrodes eurydice*	1.2Mi wg GuilfordVT, USA	GQ357190	GQ357259	GQ357324
